# Mexican Basic Psychological Need Satisfaction and Frustration Scale in Physical Education

**DOI:** 10.3389/fpsyg.2020.00253

**Published:** 2020-03-10

**Authors:** Jorge Zamarripa, René Rodríguez-Medellín, José Alberto Pérez-Garcia, Fernando Otero-Saborido, Maritza Delgado

**Affiliations:** ^1^Facultad de Organización Deportiv, Universidad Autónoma de Nuevo León, Nuevo León, Mexico; ^2^Facultad de Ciencias del Deporte, Universidad Pablo de Olavide, Seville, Spain; ^3^Facultad de Psicología, Universidad Autónoma de Nuevo León, Monterrey, Mexico

**Keywords:** self-determination theory, autonomy, competence, relatedness, physical education, invariance, gender, Mexico

## Abstract

Basic psychological needs are an energizing state that, if satisfied, will produce an increase in confidence and a healthy motivational orientation that leads to wellness. Frustration of these needs is the opposite concept of satisfaction, which refers to the negative sensation experimented by an individual when he or she perceives that their psychological needs are being actively limited by the actions of the significant other. To date, we have not found instruments validated in Spanish that measure both the satisfaction and the frustration of basic psychological needs in the physical education (PE) context. Therefore, the aims of this study are adapting the Basic Psychological Need Satisfaction and Frustration Scale (BPNSFS) to the PE context in Mexico; and examine its psychometric properties, structure, and factorial invariance by gender in a sample of fifth- and sixth-grade elementary school students. This study included a total of 1,470 fifth- and sixth-grade students from elementary schools in the metropolitan area of Monterrey, Nuevo Leon, Mexico. The results support the reliability, validity, structure, and strict invariance of the sixth Mexican version of the BPNSFS in physical education (BPNSFS-PE). The BPNSFS-PE can be used to measure the satisfaction and/or frustration of the basic psychological needs of students in PE class and to perform comparisons between groups of boys and girls.

## Introduction

The self-determination theory (SDT) (Deci and Ryan, 1985, 2002, 2014; Ryan and Deci, 2017) is a macro theory that provides a clear overview of motivational processes and their consequences in different contexts, such as education. It proposes that in individuals that develop a motivation that brings them closer to an activity (e.g., actively participate in classes), their participation in the said activity will satisfy three basic psychological needs (BNP), which are autonomy, competence, and relatedness.

*Autonomy* refers to the feeling of willfulness that can accompany any act, whether dependent or independent, collective, or individual (Ryan and Deci, 2000); in other words, it refers to the desire of the individual to be the origin of their behavior, and therefore, it is related with the freedom granted to make decisions while the activity takes place.

*Competence* refers to the ability to effectively interact with their environment to assure the conservation of the organism. Satisfaction of the need for competence provides the energy for learning (Deci and Ryan, 1985). This leads people to look for challenges that are optimal for those skills and abilities related to the activity (e.g., the skills and content of subjects); in the same way, the need for competence refers to the feeling people have to act effectively in the environment surrounding them with the aim of developing feelings of achievement.

*Relatedness* refers to being connected and respected by others (e.g., with the professor and classmates) and having a feeling of belonging to the group (Ryan, 1995).

By definition, *basic psychological needs* are an energizing state that, if satisfied, will produce an increase in confidence and a healthy motivational orientation that leads to wellness and facilitates the development of enjoyment, effort, persistence, commitment, and well-being (Deci and Ryan, 2000).

But these needs can also be decreased by a negative context. Therefore, for several years, a construct of frustration of the psychological needs has been developed by Bartholomew et al. (2011), which refers to the negative sensation experimented by an individual when he or she perceives that their psychological needs are being actively limited by the actions of a significant other (e.g., the class teacher).

The *frustration* of these needs is the opposite concept to their *satisfaction* (Bartholomew et al., 2011). For example, a student may not have a good social relationship with his or her classmates because he or she does not feel close and is not compatible with them; however, another student may not have a good relationship with his or her classmates because they reject or bother him or her. Obviously, both cases are different: the first case refers to low satisfaction of the need for a relationship, while the second is related to the frustration of this need. Thus, to value the frustration of the psychological needs, it is necessary to refer to the effect caused by the significant others in these needs.

In recent years, different instruments have been used to measure the satisfaction of basic psychological needs (BPNS) in physical education (PE), and these have been validated in different cultures, such as British (Standage et al., 2005), Spanish (Moreno Murcia et al., 2008), Brazilian (Pires et al., 2010), Greek (Vlachopoulos et al., 2011), Chinese (Liu and Chung, 2014), and Mexican (Zamarripa et al., 2017).

On the other hand, to measure the frustration of basic psychological needs (BPNF), the *Psychological Need Thwarting Scale* (*PNTS*) has been used (Bartholomew et al., 2011), which has been adapted and used in the context of PE in the Chinese (Liu and Chung, 2015) and Spanish populations (Cuevas et al., 2015, 2016).

The number of items that must be used to measure satisfaction, on the one hand, and frustration, on the other, limits the studies along these lines. Instruments that measure BPNS use a considerable number of items that vary from 16 to 33. If we add the 12 items needed to measure BPNF, the number of items needed to measure both constructs would vary between 28 and 45.

As an alternative to these instruments, Chen et al. (2015) created an instrument that measures both constructs with a smaller number of items. The instrument called the *Basic Psychological Need Satisfaction and Need Frustration Scale* (BPNSNFS) was validated with a sample in four different cultures and languages (Belgium, China, United States, and Peru). The sample consisted of 1,051 university students with a mean age of 20 years. The study authors showed evidence that the scale, composed of 24 items, presented a multidimensional structure of six factors that measure satisfaction and frustration, respectively, in each of the psychological needs. The results obtained in a cross-validation recognized four necessary items, such as an internal consistency for each dimension that ranged from 0.64 to 0.89. The model of the six factors proposed by the authors had a good fit, SBχ^2^ (231) = 441.99, CFI = 0.95, RMSEA = 0.04, and SRMR = 0.04.

Within the educational context, the PE class was one subject that was intimately linked to adopting a healthy lifestyle. In line with Telama et al. (2005), the main objective of PE in many countries must be the promotion of health in young population, and Mexico is not an exception. The high rates of sedentary and obesity in the population, in addition to a large number of children and teens that can be accessed through PE, makes this the ideal medium for promoting health and healthy fitness from an early age. For this reason, the study of the BNP in PE could contribute to achieving this objective since different studies have demonstrated that satisfying these needs leads to more positive results of physical activity; in other words, PE students that have their needs satisfied show greater commitment (Parish and Treasure, 2003), effort (Bagøien et al., 2010; Taylor and Lonsdale, 2010; Taylor et al., 2010), and amount of physical activity, as well as a better perception of quality of life related to health (Standage et al., 2012).

In this sense, Haerens et al. (2015) translated the BPNSNFS into Dutch and adapted it to the context of PE. A total of 499 students (boys = 43.8%, *M*_age_ = 15.76, *SD* = 1.16) from secondary schools in Flanders, Belgium participated. The structure of the instrument was examined with a high-order confirmatory factorial analysis (CFA) where the items were used as indicators of six first-order factors (satisfaction of autonomy, competence, and relatedness, and frustration of autonomy, competence, and relatedness), which also served as indicators of two higher-order factors, that is, BPNS and BPNF. The two-dimension model (BPNS and BPNF) had acceptable fit indexes, χ^2^ (243) = 530.49, *p* < 0.001, RMSEA = 0.05, CFI = 0.91, and SRMR = 0.06. The internal consistency of the high-order factors of satisfaction (α = 0.87) and frustration (α = 0.84) was acceptable, as well as each one of the six first-order factors (α = 0.71–0.80).

To date, we have not found instruments validated in Spanish that measure both the satisfaction and BPNF in the context of PE. The aforementioned limits the study of this area in Spanish-speaking cultures such as Mexico, and due to the importance and repercussion that the BPNS has on different aspects related to the learning and well-being of students, it becomes necessary to have reliable instruments with an adequate number of items and that measures both constructs in the context of PE. Therefore, the aims of this study are to adapt the Basic Psychological Need Satisfaction and Frustration Scale (BPNSFS) (Chen et al., 2015) to the context of PE in Mexico and examine its psychometric properties, structure, and factorial invariance by gender, in a sample of fifth- and sixth-grade Mexican elementary school students.

## Materials and Methods

### Design and Type of Study

This was a quantitative study with an instrumental design to evaluate the psychometric properties of a scale that measures the satisfaction and BPNF in the context of PE (Ato et al., 2013). This was a non-probabilistic convenience sample.

### Participants

Study participants included 1,470 students (boys = 50.6% and girls = 49.4%) from fifth (49.3%) and sixth (50.7%) grade from federal (89.3%) and state (10.7%) elementary schools of the metropolitan area of Monterrey in the morning (70.6%), afternoon (24.3%), and (5.2%) fulltime shift, with ages from 10 to 14 years (*M*_age_ = 10.56; SD = 0.77) who attended PE class twice a week with a duration of 50 min per session, and in which 68% said they practiced at least one sport outside of school. Fifth- and sixth-grade students were chosen because children who belong to the final years of third childhood and early adolescence are at the highest level of cognitive development and will not have any complications when responding to the instruments (Papalia et al., 2009).

### Instrument

To measure the students’ satisfaction and frustration, the BPNSFS (Chen et al., 2015) was translated and adapted to the PE context. The scale is composed of 24 items grouped into two factors: basic psychological needs satisfaction (BPNS) and basic psychological needs frustration (BPNF). These two factors are composed of three variables each; the BPNS is composed of satisfaction of autonomy (SA), satisfaction of competence (SC), and satisfaction of relatedness (SR). In contrast, the BPNF is composed of frustration of autonomy (FA), frustration of competence (FC), and frustration of relatedness (FR). Each one of these variables was measured by four items. The instrument has as a heading “En mi clase de Educación Física.” One example of a SA item is “…siento que tengo la libertad y posibilidad de elegir las actividades de la clase.” and of the FA “…siento que la mayoría de las actividades que hago, las hago porque tengo que hacerlas.” One example of an SC item is “…siento que puedo hacer bien las actividades.” and of FC “…tengo serias dudas acerca de que pueda hacer bien las actividades.”. One example of an SR item is “…siento que le importo a mis compañeros que me importan.” and of the FR “…me siento excluido del grupo al que quiero pertenecer.” The 24 items are answered on a Likert scale of five points that go from 1 (*Not True at All*) to 5 (*Completely True*).

### Procedure

This study was carried out according to the ethical guidelines recommended by the American Psychological Association (APA). Authorization was requested in writing from the school zone authorities and from each of the principals of the schools explaining the objectives of the research and the procedure that would be performed together with a model of the instrument. Afterward, authorization was requested for application from the teachers of each group and from the selected students taking into consideration the inclusion criteria: be a regular student in their respective group, regularly have PE class at least twice a week, be voluntarily willing to complete the questionnaire, and deliver the informed consent to participate in the research signed by their parents or tutors. The students were informed of the objective of the study, their willingness to volunteer, the absolute confidentiality of their answers, and the management of the data. They were also told that there were no correct or incorrect answers and they were asked for maximum sincerity and honesty. The questionnaire was anonymous and self-administered collectively in the classroom during school hours. To homogenize the data collection conditions, the administrators received prior preparation and training. The protocol was approved by the Ethics Committee of the Universidad Autónoma de Nuevo León (No. 16CI19039021). All subjects gave written informed consent in accordance with the Declaration of Helsinki.

The BPNSFS was translated into Mexican Spanish following the translation-back-translation procedure (Hambleton and Kanjee, 1995). The translation was carried out by a professional translation agency hired by the study team. To adapt the translation to the context of PE, a group of experts was formed with two Ph.D. specialists and with previous experience in the validation of psychological instruments, a PE teacher, and a translator specialized in the area of physical activity and sports; they discussed the translation discrepancies until the first version of the Mexican Spanish-language instrument was achieved. This version was retranslated into English by a professional translation agency different from the first and both versions were contrasted: the original and the translation. Again, the differences in the versions were analyzed and necessary changes were introduced to facilitate the comprehension of the items achieving a final version of each of the scales. This version was administered as a pilot application to a group of 72 students of different school levels to verify comprehension of each of the items; the results of this pilot application did not show any comprehension problems. The items that comprise the scale are presented in Table 1.

**TABLE 1:** Descriptive and standardized solution of the items and subscales of the instrument.

Subscales						Factorial saturations	
En mi clase de Educación Física…[In my Physical Education class…]		*M*	SD	Asymmetry	Kurtosis	Two factors	Six factors
	*Basic psychological needs satisfaction*	3.73	0.69	–0.61	0.63		
	*Autonomy satisfaction*	3.56	0.83	–0.54	0.14		
1	…siento que tengo la libertad y posibilidad de elegir las actividades de la clase. (…I feel a sense of choice and freedom in the things I undertake.)	3.19	1.40	–0.25	–1.19	0.33	0.29
7	…siento que mis decisiones reflejan lo que realmente quiero. (…I feel that my decisions reflect what I really want.)	3.69	1.17	–0.74	–0.16	0.54	0.51
13	…siento que mis elecciones expresan lo que realmente soy. (… I feel my choices express who I really am.)	3.63	1.27	–0.72	–0.48	0.61	0.57
19	…siento que he estado haciendo lo que realmente me interesa. (…I feel I have been doing what really interests me.)	3.76	1.22	–0.83	–0.26	0.63	0.61
	*Relatedness satisfaction*	3.67	0.87	–0.53	–0.06		
3	…siento que le importo a mis compañeros que me importan. (…I feel that the people I care about also care about me.)	3.70	1.22	–0.79	–0.25	0.54	0.57
9	…me siento conectado con los compañeros que se preocupan por miì y por los cuales yo me preocupo. (…I feel connected with people who care for me, and for whom I care.)	3.74	1.24	–0.81	–0.33	0.56	0.60
15	…me siento cerca y conectado(a) con otros compañeros que son importantes para mí. (…I feel close and connected with other people who are important to me.)	3.73	1.24	–0.80	–0.31	0.66	0.68
21	…tengo una sensación de calidez cuando estoy con los compañeros con los que paso tiempo. (…I experience a warm feeling with the people I spend time with.)	3.51	1.29	–0.53	–0.76	0.58	0.59
	*Competence satisfaction*	3.94	0.81	–0.79	0.47		
5	…siento que puedo hacer bien las actividades. (…I feel confident that I can do things well.)	4.15	1.05	–1.35	1.34	0.60	0.62
11	…me siento capaz en las actividades que hago. (…I feel capable at what I do.)	3.92	1.15	–1.00	0.24	0.63	0.67
17	…siento que soy capaz de alcanzar los objetivos de la clase. (…I feel competent to achieve my goals.)	3.88	1.20	–0.93	–0.06	0.64	0.66
23	…siento que puedo cumplir con éxito las actividades difíciles. (…I feel I can successfully complete difficult tasks.)	3.81	1.20	–0.88	–0.10	0.60	0.62
	*Basic psychological needs frustration*	2.75	0.81	0.02	–0.30		
	*Autonomy frustration*	2.83	0.95	–0.12	–0.54		
2	…siento que la mayoría de las actividades que hago, las hago porque tengo que hacerlas. (…most of the things I do feel like “I have to”.)	3.26	1.35	–0.37	–1.06	0.41	0.43
8	…me siento obligado(a) a hacer muchas actividades que yo no elegiría hacer. (…I feel forced to do many things I wouldn’t choose to do.)	2.73	1.37	0.16	–1.22	0.52	0.56
14	…me siento presionado(a) a hacer muchas actividades. (…I feel pressured to do too many things.)	2.58	1.38	0.38	–1.12	0.68	0.72
20	…siento que las actividades de la clase son una serie de obligaciones. (…my daily activities feel like a chain of obligations.)	2.75	1.40	0.17	–1.25	0.56	0.62
	*Relatedness frustration*	2.75	0.94	0.12	–0.62		
4	…me siento excluido del grupo al que quiero pertenecer. (…I feel excluded from the group I want to belong to.)	2.75	1.45	0.14	–1.38	0.51	0.53
10	…siento que los compañeros que son importantes para mií son fríos y distantes conmigo. (…I feel that people who are important to me are cold and distant toward me.)	2.59	1.35	0.34	–1.08	0.61	0.65
16	…tengo la impresión de que le disgusto a los compañeros con los que paso tiempo. (…I have the impression that people I spend time with dislike me.)	2.67	1.35	0.29	–1.11	0.62	0.65
22	…siento que la relación con mis compañeros es superficial. (…I feel the relationships I have are just superficial.)	2.98	1.34	–0.09	–1.15	0.49	0.51
	*Competence frustration*	2.68	0.98	0.14	–0.70		
6	…tengo serias dudas acerca de que pueda hacer bien las actividades. (…I have serious doubts that I can do the activities well.)	3.14	1.38	–0.25	–1.18	0.51	0.51
12	…me siento decepcionado(a) con muchas de mis participaciones. (…I feel disappointed with many of my performance.)	2.57	1.35	0.35	–1.11	0.66	0.67
18	…me siento inseguro(a) de mis habilidades. (…I feel insecure about my abilities.)	2.60	1.38	0.31	–1.20	0.69	0.70
24	…me siento como un(a) fracasado(a) por los errores que cometo. (…I feel like a failure because of the mistakes I make.)	2.41	1.42	0.53	–1.09	0.61	0.62

*All saturations were significant, *t* > 1.96, *p* < 0.05*

### Data Analysis

First, a descriptive analysis was performed for all the scales and the factors that comprise them. To test the factorial structure of the questionnaire, a confirmatory factor analysis (CFA) was performed of the two proposed models (of two and four factors). Taking into consideration its ordinal nature, the sample size, the number of response options (*k* = 5), and the symmetry and kurtosis values of the items (see Table 1), the CFA was performed with the *maximum likelihood* (ML) method and as output the asymptotic covariance matrix of the polychoric correlations was used.

Model adequacy was analyzed with different fit indexes, such as the CFI, NNFI, and RMSEA. CFI and NNFI values greater than or equal to 0.95 indicate an acceptable fit (Hu and Bentler, 1999). For RMSEA, negative values or equal to or lower than 0.08 are considered satisfactory (Cole and Maxwell, 1985).

To determine which of the two models (two and six factors) adjusted better to the data, the differences between the goodness-of-fit indexes of the models were analyzed. Differences no greater than 0.01 between the CFI and NNFI values (Widaman, 1985; Cheung and Rensvold, 2002) and of 0.015 between the RMSEA values (Chen, 2007) were considered irrelevant in the models comparison, and therefore, claim support for the more constrained (parsimonious) model.

To determine if the Mexican version of the BPNSFS in physical education (BPNSFS-PE) shows invariance by gender, a multigroup CFA was performed. Incremental goodness-of-fit indexes of the alternative models were estimated. A difference of 0.01 or less between the CFI (Cheung and Rensvold, 2002) and of 0.05 between the NNFI values (Little, 1997) reflects practically irrelevant differences between the models. Regarding the RMSEA, a value of 0.015 or less between the alternative models indicates irrelevant differences (Chen, 2007).

Internal consistency of the instruments was evaluated with Cronbach’s alpha (Cronbach, 1951); also, Pearson’s correlation analysis was performed between all the variables. These were carried out using the statistical package SPSS Statistics V.21 and the program LISREL 8.80 (Jöreskog and Sörbom, 2006).

## Results

### Descriptive Analysis and Normality

The descriptive analysis (mean, standard deviation, asymmetry, and kurtosis) of each of the items, variables, and factors that compose the scale is shown in Table 1. The results reveal that BPNS values are higher than the BPNF in the PE class. Particularly, competence is the psychological need that had the highest satisfaction values. On the other hand, autonomy was the psychological need that had the highest frustration values. Most of the asymmetry and kurtosis values were outside the range (−1.5 to 1.5), indicating a normal distribution of data (Shumacker and Lomax, 2004).

### Confirmatory Factorial Analysis and Model Comparison

The goodness-of-fit of the two- (SBχ^2^/*df* = 5.112, NNFI = 0.951, CFI = 0.955, and RMSEA = 0.053) and six-factor model (SBχ^2^/*df* = 4.94, NNFI = 0.953, CFI = 0.960, y RMSEA = 0.052) was satisfactory. The values obtained from the differences between the fit indexes of both were irrelevant (ΔNNFI = 0.002, ΔCFI = 0.005, y ΔRMSEA = 0.001), which suggests that both models fit in a similar way; therefore, a more parsimonious model should be selected, in this case, the two-factor model (see Table 2). All estimated factorial saturations for the two- and six-factor model were significant (see Table 1).

**TABLE 2:** Goodness of fit indices of the confirmatory factor analysis of the two proposed models.

Models	χ^2^/*df*	RMSEA	ΔRMSEA	CFI	ΔCFI	ΔNNFI	ΔNNFI
Two factors	5.11	0.053		0.955		0.951	
Six factors	4.94	0.052	0.001	0.960	0.005	0.953	0.002

**df*, degrees of freedom; RMSEA, root mean square error of approximation; NNFI, non-normed fit index; CFI, comparative fit index.*

### Reliability

The results of the reliability analysis revealed alpha values of 0.55–0.66 for the SA, SR, and SC. This situation was similar to those of the FA, FR, and FC, which had alpha values of 0.62–0.66. Nevertheless, the internal consistency of the scales that measure BPNS and BPNF as a global measure presented good reliability with alpha values of 0.81 and 0.83, respectively.

### Correlation Between Factors

The Pearson correlation analysis between the study variables revealed that BPNS had strong positive and significant correlations with SA, SR, and SC, and weak negative correlations with BPNF and FC. On the other hand, BPNF had strong positive and significant correlations with FA, FR, and FC; weak with SA; and negative with SC and SR (see Table 3).

**TABLE 3:** Bivariate correlations and internal consistency of the all variables of study.

	α	1	2	3	4	5	6	7
1. BPN. S	0.81	1						
2. Autonomy satisfaction	0.55	0.82**	1					
3. Relatedness satisfaction	0.65	0.85**	0.53**	1				
4. Competence satisfaction	0.66	0.83**	0.51**	0.58**	1			
5. BPN. F.	0.83	−0.06*	0.05*	−0.08**	−0.14**	1		
6. Autonomy frustration	0.64	–0.05	0.06*	−0.05*	−0.12**	0.83**	1	
7. Relatedness frustration	0.62	–0.02	0.06*	–0.04	−0.08**	0.84**	0.52**	1
8. Competence frustration	0.66	−0.10**	0.01	−0.10**	−0.15**	0.87**	0.58**	0.63**

S, satisfaction; F, frustration; BPN, basic psychological needs; α, Cronbach alpha; *p < 0.05; **p < 0.01.

### Measurement Invariance

Based on the results of the CFA, invariance was evaluated based on the gender of the two-factor model. A preliminary analysis was performed that separately examined the structure of the BPNSFS-PE in the sample of boys (Model M0a) and girls (Model M0b). As shown in Table 4, the goodness-of-fit indexes of the models M0a and M0b were satisfactory with all the estimated parameters being statistically significant (*p* < 0.01).

**TABLE 4:** Goodness of fit indexes of invariance models.

Model	Model description	*df*	SBχ^2^	RMSEA	90% CI	NNFI	CFI	ΔNNFI	ΔCFI	ΔRMSEA
M0a	Baseline model boys	251	815.88**	0.055	0.051–0.059	0.950	0.955			
M0b	Baseline model girls	251	737.67**	0.052	0.048–0.056	0.950	0.955			
M1	Structural invariance (baseline model)	503	1554.54**	0.053	0.050–0.056	0.950	0.955			
M2	FL invariance	526	1694.45**	0.055	0.052–0.058	0.947	0.950	0.003	0.005	0.002
M3	FL + INT invariance	548	2009.23**	0.060	0.058–0.063	0.937	0.937	0.013	0.018	0.007
M4	SF + Inv. + Error invariance	572	2305.58**	0.064	0.062–0.067	0.928	0.925	0.022	0.03	0.011

*df*, degrees of freedom; RMSEA, root mean square error of approximation; 90% CI, 90% confidence interval for the RMSEA; NNFI, non-normed fit index; CFI, comparative fit index; Inv., Invariance; FL, factor load; INT, intercepts. All comparisons in the Δ indices are made with respect to the baseline model (M1). **p < 0.01.

Later, a multisample analysis was carried out. Model 1 (M1) examined the structural invariance of the BPNSFS-PE in both of the analyzed groups showing that the goodness-of-fit was satisfactory; therefore, we concluded that the factorial structure of the instrument is invariant in the two compared groups (see Table 4). The M1 was used as the basis for the nesting of restrictions.

Model 2 (M2), which tested the equivalence of factor saturations across the group of boys and girls, showed adequate fit indexes. After comparing these indexes with those of M1, the differences did not exceed the criteria values (ΔCFI < 0.01 and ΔNNFI < 0.05; ΔRMSEA < 0.015); therefore, this work presents evidence of the invariance of the factorial saturations of BPNSFS-PE across the evaluated sample.

Model 3 (M3) or the “strong factorial invariance model” (Meredith, 1993), which adds the equivalence of the intercepts, showed satisfactory goodness-of-fit indexes. The values obtained from the differences between the NNFI and the RMSEA from M3 and M1 did not exceed the criterion values. However, this did not happen with the CFI (see Table 4); nevertheless, it can be concluded that the equivalence of factorial saturations and intercepts can be accepted when the invariance is fulfilled for two parameters, although not for the CFI.

Finally, Model 4 (M4) or the “strict factorial invariance model” (Meredith, 1993), which adds invariance to the factorial saturations, intercepts, and errors, also presented satisfactory fit indexes. As in the previous comparison, the difference obtained between the NNFI and the RMSEA of the M4 and M1 did not exceed the criterion values, except for the CFI; however, it can be concluded that the strict factor equivalence of the BPNSFS-PE is accepted across gender when the invariance in two of the three parameters examined is fulfilled.

## Discussion

The aims of this study were to adapt the BPNSFS (Chen et al., 2015) to the context of PE in Mexico and examine its psychometric properties, structure, and factorial invariance by gender, in a sample of fifth- and sixth-grade Mexican elementary school students.

The results of the CFA revealed a good fit of the data for the two proposed models (two and six dimensions). Regarding the two-dimensional model (BPNS and BPNF), our results were consistent with those obtained from the Dutch translation adapted to PE by Haerens et al. (2015). Likewise, the goodness-of-fit indexes of the six-factor model (SA, SR, SC, FA, FR, and FC) found in this study were also consistent with the satisfactory fit of the PE version used by Cuevas et al. (2018) and in the version for the general context developed by Chen et al. (2015).

Regarding model comparison (two and six factors), the product obtained from the incremental differences of both models did not exceed the criterion values; this indicates irrelevant differences between both models; therefore, the most parsimonious model was selected, that is, the two-factor model for the invariance analysis. These results differ from those found by Cordeiro et al. (2016), where the six-factor model presented better goodness-of-fit indexes that coincide with those found by Chen et al. (2015). However, the instrument used in both studies measured need satisfaction and frustration in a general context (instead of a specific domain). Nevertheless, within the context of PE, the results of the study by Haerens et al. (2015) coincide with this work using the two-factor model (satisfaction and frustration).

The internal consistency of the factors that correspond with satisfaction (SA, SR, and SC) and frustration (FA, FR, and FC) did not reach the criterion value recommended by some authors (Nunnally and Bernstein, 1994; Bland and Altman, 1997). Schmitt (1996) has suggested that there is no general level (e.g., 0.70) in which the alpha becomes “acceptable,” but the instruments with a very low alpha can still be useful in some circumstances, for example, when a scale is composed of a small number of items (Dall’oglio et al., 2010) and in the first stages of studies such as in this work (Nunnally, 1967). However, the internal consistency of the scales that measure BPNS and BPNF presented adequate reliability, even without reaching the maximum acceptable alpha, since according to Streiner (2003), instruments with high values (e.g., >0.90) could suggest that the items are redundant and that they are measuring the same question but in a different way.

The results of the correlation analysis between dimensions (BPNS and BPNF) and their respective factors (SA, SR, SC, FA, FR, and FC) revealed positive and significant relatedness between them, as well as a negative and significant correlation between BPNS and BPNF. These correlations similarly coincide with the results reported in other studies that have used the same instrument (Chen et al., 2015; Haerens et al., 2015; Nishimura and Suzuki, 2016; Cuevas et al., 2018).

Finally, the results of the multigroup CFA revealed a strict factorial invariance of the BPNSFS-PE across sex in the two-factor structure. No recent studies have been found that examine the invariance of this instrument through gender groups in the context of PE. In the general context, the results of the study by Tóth-Király et al. (2018) revealed invariance by gender in the Hungarian version of the instrument; however, this analysis was performed on a model composed of two factors (BPNS and BPNF) and on a global one called *global need fulfillment*, which differs from the model we propose.

## Conclusion

After examining the psychometric properties, structure, and factorial invariance of the Mexican version of the BPNSFS in the context of PE (BPNSFS-PE), it can be concluded that it is a reliable and valid instrument that can be used to measure the satisfaction and/or frustration of students’ BPNs in PE class and make comparisons between groups of boys and girls, either as a two-factor (BPNS and BPNF) or a six-factor (SA, SR, SC, FA, FR, and FC) model according to the research purpose and question of each study, in order to increase the generation of knowledge and scientific production of this area in Mexico, since its factorial structure coincides with that used in previous studies and it is consistent with the assumptions of the SDT (Deci and Ryan, 1985, 2002, 2014; Ryan and Deci, 2017).

This study also has some limitations. The study participants only include fifth- and sixth-grade students from elementary schools in the metropolitan area of Monterrey; therefore, future research should include population from different school levels and sectors in the country. In addition, the study of the psychometric properties of the instrument could be expanded to include population from other Spanish-speaking countries, and in this way, contribute to the conduction of cross-cultural studies. Lastly, we suggest including in the study of factorial invariance the educational grades and levels, areas and populations of other sectors of the country, as well as population from different Spanish-speaking countries to determine its function and facilitate the comparison of results.

## Practical Applications

This instrument can be used by teachers, school principals, institutions responsible for PE, and researchers to perform studies with Mexican population with the aim of knowing the levels of satisfaction and frustration of students during PE class and to make comparisons between boys and girls. The aforementioned is of vital importance for learning because, as mentioned before, when needs are satisfied, there is an increase in confidence and a healthy motivational orientation that leads to health and that facilitates the development of enjoyment, effort, persistence, commitment, and well-being (Deci and Ryan, 2000).

## Data Availability Statement

The datasets generated for this study are available on request to the corresponding author.

## Ethics Statement

The studies involving human participants were reviewed and approved by Ethics Committee of the Universidad Autónoma de Nuevo León (No. 16CI19039021). Written informed consent to participate in this study was provided by the participants’ legal guardian/next of kin.

## Author Contributions

JZ, RR-M, and FO-S conceived the hypothesis of this study. JP-G and MD participated in data collection and methodology. JZ and MD analyzed the data. JZ and RR-M wrote the manuscript with significant input from JZ. JZ took charge of funding acquisition. All authors contributed to data interpretation of statistical analysis, and read and approved the final manuscript.

## Conflict of Interest

The authors declare that the research was conducted in the absence of any commercial or financial relationships that could be construed as a potential conflict of interest.
